# Kinesio Taping as an Adjunctive Nursing Intervention for Lower Extremity Edema in ICU Patients: A Case Series

**DOI:** 10.3390/reports9020158

**Published:** 2026-05-19

**Authors:** Yeshua Aguilar-Salgado, Antonio Hernández-Bastida, María de la Paz Lara-Martínez, Blanca Estela García-Pérez, Lorena García-Morales, Alejandra Valdivia-Flores

**Affiliations:** 1 Departamento de Posgrado, Escuela Superior de Enfermería y Obstetricia, Instituto Politécnico Nacional, Ciudad de México 11340, Mexico; yeshuamiji@gmail.com (Y.A.-S.); antoniohernandezbastida@hotmail.com (A.H.-B.); mlara@ipn.mx (M.d.l.P.L.-M.); logarciam@ipn.mx (L.G.-M.); 2 Dirección del Hospital Obregón, Hospital Obregón, Ciudad de México 06800, Mexico; 3 Servicio de la Terapia Médica Intensiva Central, Hospital General de México “Dr. Eduardo Liceaga”, Ciudad de México 06720, Mexico; 4 Departamento de Microbiología, Escuela Nacional de Ciencias Biológicas, Instituto Politécnico Nacional, Ciudad de México 11340, México; abrilestela@hotmail.com; 5 División de Investigación, Hospital Juárez de México, Ciudad de México 07760, México

**Keywords:** Kinesio tape, edema, intensive care unit

## Abstract

**Background and Clinical Significance**: Kinesio tape (KT) has gained popularity as an adjunctive approach for treating edema during the rehabilitation phase, following traumatic events, as well as for managing edema in breast cancer patients. Its goal is to reduce swelling and improve mobility in the affected extremity; however, its use in critically ill patients remains limited. To our knowledge, this is the first report of its application in this population. **Case presentation**: This case series involved three patients in the Intensive Care Unit (ICU) who presented with lower extremity edema. One patient developed a cerebrovascular event secondary to moderate traumatic brain injury and two patients experienced sepsis. KT was applied, and extremity circumference, Godet sign, and Stemmer sign were assessed. The bandage was reapplied every 24 h over a 5-day period, with daily evaluations performed by the same nursing staff to ensure measurement consistency. All three patients exhibited a reduction in extremity circumference, along with improvement or resolution of the Godet and Stemmer signs. No adverse effects associated with KT were observed. **Conclusions**: Our results suggest that KT may be a beneficial adjunctive therapy for edema reduction in ICU patients. Larger-scale studies are needed to confirm its clinical value.

## 1. Introduction and Clinical Significance

The prevalence of lower extremity edema among patients in Intensive Care Units (ICUs) is significantly high, affecting between 20% and 50% of patients [1]. In the ICU setting, this condition is often multifactorial, resulting from prolonged immobility, the physiological effects of mechanical ventilation on venous return, and systemic inflammatory responses. Furthermore, common comorbidities such as cardiac dysfunction, renal failure, and hypoalbuminemia contribute to fluid extravasation and edema formation [2,3]. Its management requires a comprehensive approach that address each patient’s underlying pathological conditions and encompasses both surgical and non-surgical strategies. Surgical interventions are typically reserved for chronic obstructive cases (lymphedema) and aim to improve lymphatic transport through physiological procedures, such as lymphaticovenular anastomosis, or reductive techniques like suction-assisted protein-carving lipectomy [4]. In contrast, non-surgical strategies remain the cornerstone of conservative management. These include manual lymphatic drainage, compression therapy, and the application of Kinesio Tape (KT), which provide a non-invasive means to facilitate interstitial fluid drainage and reduce extremity volume [5].

Kinesio taping has been reported to contribute to pain relief and to enhance blood and lymphatic circulation by promoting localized lifting of the skin, which may facilitate lymphatic drainage toward regional lymph node basins. This mechanism can help reduce interstitial pressure and support lymphatic system function while influencing the skin, fascia, muscles, joints, and vascular structures [6].

KT has been used as an adjunctive therapy in the management of edema during the musculoskeletal rehabilitation phase, contributing to the reduction in swelling, alleviation of pain, and improvement of mobility in affected areas, including the face and extremities [5,7,8]. Greater benefits have been reported when KT is combined with manual lymphatic drainage, therapeutic exercise, extremity mobilization, and skin care [9]. Reported treatment durations range from 24 h to 4 weeks, and no adverse effects have been documented to date [5]. KT has also been evaluated in the treatment of traumatic and postoperative vascular edema [10] as well as in the management of cancer-related upper-extremity lymphedema [11] with favorable outcomes. Moreover, KT has shown comparable results to multilayer bandaging in the treatment of edema in breast cancer patients, with additional advantages such as being less invasive, allowing unrestricted mobility, and reducing treatment costs [12].

In Intensive Care Units (ICUs), the management of lower extremity edema presents a significant challenge. To date, the application of Kinesio Tape in critically ill patients with lower extremity edema has not been explored as a potential nursing intervention. This study aims to explore this application.

Clinical Significance: This case series explores the use of Kinesio tape as a complementary approach in the management of lower extremity edema in critically ill patients. The findings suggest a reduction in edema associated with the underlying condition, supporting its potential role as an adjunctive therapy for edema management in the ICU.

## 2. Case Presentation

Following institutional ethics committee approval (No. 2024-017) and the acquisition of written informed consent from all patients or their relatives, three critically ill patients were selected. All participants were clinical candidates for compression therapy to manage lower extremity edema. Inclusion criteria required adult ICU patients to exhibit clinically evident lower extremity edema and maintain hemodynamic stability that allowed extremity manipulation. Conversely, patients were excluded if they required vasopressor support, presented skin lesions or local infections, had a history or signs of deep vein thrombosis (DVT), or showed decompensated pulmonary, cardiogenic, or renal edema. Additionally, known hypersensitivity to adhesives or severe dermal fragility served as exclusion criteria.

Edema was assessed through circumference measurements and clinical evaluation using Stemmer’s and Godet’s signs to determine the presence and severity of tissue swelling. Each patient underwent an initial edema assessment that included extremity circumference measurements at three segments, evaluation of the Godet’s and Stemmer’s signs, and collection of laboratory data. After cleaning the treatment area, a comprehensive evaluation was performed, considering mobility, skin color, Godet and Stemmer signs, hydration, skin integrity, capillary refill, temperature, urine output, and 24-h fluid balance. Edema assessment included the evaluation of pitting, tenderness, and skin alterations. The Godet sign, or pitting edema, is characterized by a persistent indentation in edematous tissue following the application of finger pressure, indicating interstitial fluid accumulation [3]. This sign was assessed by documenting its location, depth, and recovery time to classify the severity of edema. It was graded on a scale of 1+ to 4+. Additionally, we assessed the Stemmer sign, which is determined by the inability to pinch and lift a skin fold at the base of the second toe or finger duo to dermal thickening and fibrosis [13]. A positive Stemmer sign is a pathognomonic indicator of chronic lymphedema and skin remodeling.

KT was applied using the “octopus” technique, also known as the “fan” technique, and according to the placement manual [6], performed by personnel trained and certified in its application. This technique was specifically selected for its efficacy in lymphatic drainage. This configuration consists of narrow, fan-shaped strips that overlap the edematous area, applied with light tension (15–25% stretch). The therapeutic rationale for this choice lies in its ability to create tegumentary convolutions that lift the skin, thereby reducing interstitial pressure and facilitating the opening of initial lymphatic vessels. The base (anchor) of the tape is positioned proximally near functional lymph nodes to provide directional pathway for fluid mobilization, while the active strips are distributed over the area of maximum accumulation [14]. The bandage was replaced every 24 h for 5 consecutive days, with daily assessments of the extremity to rule out medical adhesive-related skin injury (MARSI) or other complications. Finally, circumference measurements of the designated extremity segments were repeated three times at every occasion, until completion of the 5-day treatment.

### 2.1. Case Report 1

A 60-year-old female patient with an ectomorphic body type, weighing 56 kg and measuring 1.55 m in height, with a Body Mass Index (BMI) of 23.3, was admitted with a diagnosis of septic shock secondary to soft tissue infection. She reported having undergone left hip surgery one year earlier, which subsequently became infected. Despite outpatient management with analgesics, antibiotics, and wound care, the infection did not resolve, leading to hospital admission and surgical debridement.

She subsequently required vasopressor support and invasive mechanical ventilation. Within 24 h, weaning from both supports was initiated and successfully completed. The lower left extremity showed marked edema, with positive Godet (+++++) and Stemmer signs. The right extremity had pressure injuries and was excluded from intervention. At the time, edema was being managed with elastic bandages; following the initial assessment, KT was applied.

The patient’s daily vital signs remained stable, with normal heart and respiratory rates and normothermia except for a fever on day three. Oxygenation goals were maintained with 2 L/min via face mask, and blood pressure remained within target ranges. Blood glucose levels were acceptable, and laboratory results showed mild hyponatremia, otherwise normal electrolyte levels, and hypoalbuminemia (Table A1).

**Figure 1:**
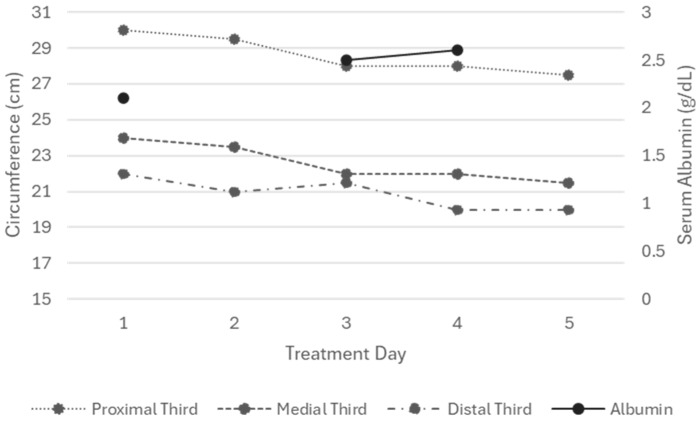
Circumference measurements of the proximal, middle, and distal thirds of the lower left extremity (Patient 1). All values represent the mean of three consecutive measurements.

The application of KT for edema management resulted in a reduction in extremity circumference at the end of the intervention. The distal third (DT) measured 24 cm at baseline and 21.5 cm at the end of treatment. The medial third (MT) decreased from 22 to 20 cm, and the proximal third (PT) from 30 to 27.5 cm (Figure 1).

The skin condition was considered optimal for KT, with no external factors that could predispose injury. The skin remained intact, adequately hydrated, and showed localized ecchymosis in the tibialis anterior region. No Medical Adhesive-Related Skin Injuries (MARSI) or inflammatory signs were observed; however, a tendency toward skin pallor was noted during bandage application. Edema showed the greatest improvement after three days of treatment with KT, resulting in a negative Stemmer sign by the end of the intervention, along with a partial reduction in the Godet sign from (++++) to (++), and an improvement in capillary refill time from 4 to 3 s (Table A2).

Overall, the treated extremity remained cold despite being covered and the patient being normothermic. Fluid balance was positive but within acceptable parameters, with continuous mobilization and adequate urine output. The feet were not maintained at a 30° elevation. The patient did not exhibit fluid overload, and renal function, as assessed by urine output, was preserved (Table A3).

### 2.2. Case Report 2

This case involved a 51-year-old female patient with an endomorphic body type, weighing 81 kg and measuring 1.67 m in height, with a BMI of 29.1 who developed septic shock secondary to complicated grade IV appendicitis. She underwent partial colectomy and abdominal lavage, after which she required vasopressors support and mechanical ventilation. Both supports were successfully withdrawn within 36 h through weaning and titration. On examination, the lower extremities were intact, with a positive Godet sign (+++) and a negative Stemmer sign. The intervention was initiated following the initial assessment.

The patient maintained a stable heart rate, with a slightly elevated respiratory rate and normal temperature. Oxygenation goals were achieved with 3 L/min via nasal cannula, and blood pressure remained stable without vasopressors. Blood glucose stayed within normal limits. Laboratory results showed persistent hyponatremia, corrected hypokalemia, normal chloride and calcium levels, normal magnesium levels on day 3, no available phosphorus data, and ongoing hypoalbuminemia (Table A4).

**Figure 2:**
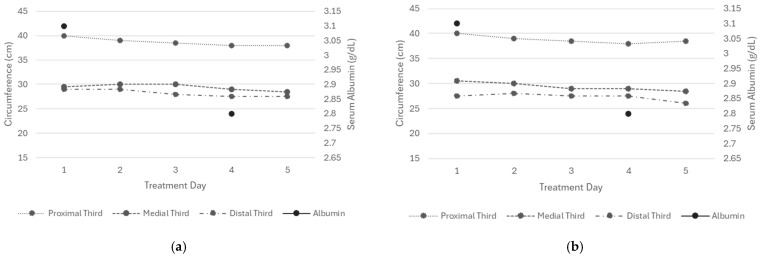
Circumference measurements of the proximal, middle, and distal thirds of both lower extremities (Patient 2). All values represent the mean of three consecutive measurements. (**a**) Right extremity and (**b**) left extremity.

Edema in the right lower extremity showed a reduction after five days of treatment. DT decreased from 29 to 27.5 cm, MT from 29.5 to 28.5 cm, and PT from 40 to 38 cm (Figure 2, left). In the left extremity, measurements decreased from 27.5 to 26.5 cm in DT, from 30.5 to 28.5 cm in MT, and from 40 to 38.5 cm in PT (Figure 2, right).

The skin of the treated extremities remained intact, hydrated, and free of ecchymosis, MARSI lesions, or inflammation, though mild redness was more noticeable during tape application. Daily edema assessments showed a negative Stemmer sign, with the most evident improvement on day three. The Godet sign improved from (+++) to (++), while capillary refill time stayed at 3 s (Table A5).

The extremities remained warm, with a positive fluid balance and continuous mobilization without maintaining the lower extremities at 30° elevation. The patient did not experience fluid overload, and renal function, as assessed by urine output, remained preserved (Table A6).

### 2.3. Case Report 3

This case describes a 56-year-old overweight female patient (BMI 29.7) admitted with a hemorrhagic cerebrovascular event following moderate traumatic brain injury. She arrived with a Glasgow Coma Scale score of 9 and was placed on invasive mechanical ventilation for neurological protection, without requiring vasopressors. After 48 h, she was evaluated for inclusion in the study. Both lower extremities were intact, showing a positive Godet sign (++) and a negative Stemmer sign.

The patient maintained a stable heart rate and a respiratory rate well synchronized with the mechanical ventilator settings. She remained normothermic, with a ventilator oxygen flow set at 40%, maintaining oxygenation targets. Blood pressure remained stable without vasopressor support, and blood glucose levels within accepted parameters. Daily serum electrolyte and albumin measurements indicated adequate metabolic management, although a decrease in serum albumin was observed (Table A7).

**Figure 3:**
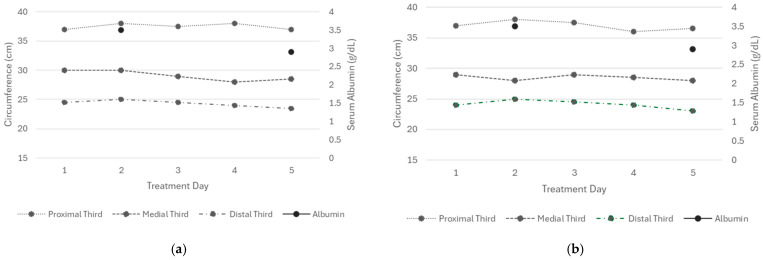
Circumference measurements of the proximal, middle, and distal thirds of both lower extremities (Patient 3). All values represent the mean of three consecutive measurements. (**a**) Right extremity and (**b**) left extremity.

In the right lower extremity, a reduction in circumference was observed by the fourth day of treatment, following an initial increase. DT decreased from 24 to 23 cm, MT from 29 to 28 cm, and PT from 37 to 36.5 cm (Figure 3, left). In the left extremity, reductions were also observed on the fourth day of treatment: DT decreased from 24.5 to 23.5 cm, MT from 30 to 28.5 cm, and PT remained at 37 cm at the end of treatment (Figure 3, right) despite the marked decrease in albumin compared with levels at hospital admission.

The skin of the treated extremities remained intact, well-hydrated, and free of ecchymosis, MARSI lesions, or signs of inflammation, although mild redness was noted during tape application. Edema assessment showed a negative Stemmer sign, with the most notable improvement after day four. The Godet sign remained stable at (++), and capillary refill time stayed at 2 s (Table A8).

Finally, the extremities were warm to the touch, with a negative fluid balance observed during the last days. The patient underwent continuous mobilization, with the lower extremities positioned at a 30° angle and maintained adequate urine output. She did not experience fluid overload, and renal function, as assessed by urine output, remained preserved (Table A9).

## 3. Discussion

The management of edema in critically ill patients in the ICU generally involves treating the underlying condition, mobilizing the patient, and using compression strategies when clinically appropriate [15]. In this context, our findings provide preliminary clinical evidence on the potential role of KT as a complementary and adjunctive intervention in critically ill patients. In our case series, which included two patients with sepsis and one with hemorrhagic cerebrovascular disease secondary to trauma, a progressive reduction in extremities circumference was observed over five days following the application of KT. This consistent trend across patients with different underlying conditions suggests a possible beneficial effect of this intervention in heterogeneous ICU populations. While KT has been widely used in sports rehabilitation for the management of inflammation, edema, and hematomas, and has shown favorable results in postoperative settings [16,17,18,19], to our knowledge, no previous reports have evaluated its effects in critically ill patients. Therefore, our results expand current knowledge by introducing a potential new application of this technique in the intensive care setting.

In our study, patient 3 showed an increase in extremity circumference in both extremities during the first three days. Nonetheless, by comparing blood pressure, albumin levels, and fluid balance, we determined that there was initial fluid overload. With treatment, edema fluid was redistributed, affecting the measurements of the different extremity segments. By the end of the intervention, a consistent reduction was observed in all segments of the extremities.

In the patient with a cerebrovascular event secondary to moderate traumatic brain injury, a differentiated evolution of edema was observed compared to septic shock patients included in the study. During the first three days of KT application, both lower extremities exhibited an increase in circumference. When correlated with blood pressure, serum albumin levels, and fluid balance, this finding was explained by an initial fluid overload. From a pathophysiological standpoint, hypoalbuminemia reduces plasma oncotic pressure, favoring fluid movement into the interstitial space. Combined with excess intravascular volume, this condition delays the response to compressive interventions. Once the fluid imbalance was compensated, treatment promoted redistribution of interstitial fluid, leading to a progressive reduction across all measured segments of both lower extremities [20].

In contrast, septic shock patients demonstrated an earlier and more homogeneous reduction in extremity circumference. This difference may be explained by the pathophysiology of sepsis, where edema is primarily associated with increased capillary permeability and endothelial dysfunction—mechanisms that tend to respond more rapidly to external compression and hemodynamic support. Previous studies have reported that hypoalbuminemia and fluid overload are limiting factors in the resolution of peripheral edema [21], consistent with the present findings.

A potential explanation for the differential response observed between patients in our series may lie in the underlying pathophysiology of edema and the patterns of fluid balance. In the patient with sepsis, a persistently positive fluid balance suggests a relatively stable state of fluid overload, likely driven by increased capillary permeability and subsequent interstitial fluid accumulation. Under these conditions, KT may enhance lymphatic and venous drainage by increasing subcutaneous space, thereby facilitating fluid mobilization and contributing to a more consistent reduction in limb circumference.

In contrast, the patient with post-traumatic intracranial hemorrhage exhibited a fluctuating balance, alternating between positive and negative states. This variability likely reflects dynamic changes in intravascular volume, tissue perfusion, and fluid redistribution, resulting in a less stable pattern of edema formation. Moreover, neurogenic and vascular alterations associated with brain injury may further disrupt peripheral circulation and fluid regulation. Together, these factors may account for the less consistent and initially paradoxical response to KT observed in this case.

Taken together, these findings suggest that the effectiveness of KT is influenced not only by the presence of edema but also by its underlying mechanisms and the temporal stability of the patient’s fluid status. In this regard, the clinical effectiveness of compressive strategies, including Kinesio taping, appears to be modulated by each patient’s pathophysiological profile. In patients with hypoalbuminemia or fluctuating fluid balance, treatment response may be delayed or exhibit transient worsening, whereas in patients with a more stable pattern of fluid overload, such as those with sepsis, edema resolution may follow a more predictable course.

These observations underscore the importance of tailoring therapeutic interventions to the hemodynamic and metabolic context of critically ill patients, highlighting the need for individualized approaches when implementing adjunctive strategies for edema management in the ICU.

These results suggest that the clinical effectiveness of compressive strategies, whether Kinesio tape or elastic stockings, may be modulated by each patient’s underlying pathophysiological profile. In individuals with hypoalbuminemia or positive fluid balance, treatment response may initially be slower or even paradoxical (transient edema increase), whereas in septic shock patients, edema resolution tends to be more uniform. This underscores the importance of tailoring therapeutic approaches to the hemodynamic and metabolic status of the patient.

Furthermore, a previous meta-analysis [22] reported statistically significant positive effects after five days of application, with greater improvement observed when treatment was extended to 4–6 weeks for musculoskeletal injuries. Although our results show a reduction in edema of the lower extremities, they are consistent with those reported by Alcántara et al. [5] regarding the need for further studies. Their review indicates that current evidence remains of low quality, even though KT has shown some superiority over comparators such as current best practice, manual lymphatic drainage, placebo and splint in follow-up periods of 10 days or less. Furthermore, for the management of acute edema, KT appears to remain effective in long-term treatment when compared to usual care, placebo and compression stockings [5].

Overall, we found that the application of KT may directly contribute to the resolution of the Stemmer sign and the reduction in the Godet sign, which could affect capillary refill and consequently decrease edema. It is important to consider that the patient’s critical condition influences several factors that determine the application of KT. Therefore, a short, five-day intervention was implemented, with daily monitoring of the treated extremities [23] as well as other clinical parameters that could indicate changes in the patient’s critical status or skin integrity, and no alterations were observed in any of the cases studied. The underlying pathology for which the patient is in the ICU, the length of hospital stays, and the duration of edema development must also be considered [24], along with appropriate management to prevent pressure injuries.

It is important to note that the high prevalence of Deep Vein Thrombosis (DVT) in ICU patients significantly restricts the application of compression therapy in the lower extremities [25]. In contrast, upper extremity edema management carries a lower risk of major embolic events due to direct lymphatic drainage. In this case series, the absence of vasopressor support and negative DVT screening were fundamental safety filters, ensuring that the mechanical mobilization of fluid did not compromise patient integrity.

The application of KT must be performed by a trained healthcare professional, certified and continuously updated, to ensure correct placement and effective functioning of the tape [14]. The procedure is also considered costly if the tape is changed daily; however, daily skin assessments did not reveal any dermal injuries. This finding suggests the possibility of future studies evaluating longer application periods without daily changes, which could reduce costs.

In critically ill patients, the use of KT as an adjunct in edema management should be indicated with caution. It is contraindicated in the presence of skin lesions, local infections, capillary fragility, suspected deep vein thrombosis, or hypersensitivity to adhesives, conditions frequently encountered in the ICU. Likewise, caution is advised in cases of hemodynamic instability and edema of multifactorial origin, where its effect may be limited if the underlying cause is not addressed. Monitoring skin integrity is essential to prevent complications. In this context, individualized patient assessment is central to its safe use, and it should always be considered a complementary intervention adapted to the patient’s overall clinical condition.

Although overall clinical improvement may contribute to edema reduction, in critically ill patients, edema is often multifactorial and may persist despite control of the underlying disease. In our case series, edema reduction was observed in temporal association with the application of KT, even in the absence of complete recovery from the primary condition, suggesting a possible local adjuvant effect. However, it must be acknowledged that no statistical correlation analysis was performed due to the small sample size (*n* = 3); therefore, the observed relationship remains strictly descriptive.

This study highlights the need for further research in this field due to the fragile health status of critically ill patients. Moreover, there is a need for continued evidence-based investigations with greater scientific rigor, considering the specialized application of the tape and the various pathologies that may influence the development of lower extremity edema in Intensive Care Units.

In addition to the quantitative outcomes observed in the reduction in extremity circumference in patients treated, they also reported enhanced sensory comfort and a decrease in subjective painful discomfort, providing a less restrictive management alternative compared to traditional compression therapies, as mentioned in a previous systematic review and meta-analysis in which the effects of lower extremity KT on pain, strength, and balance following fatigue was evaluated [26].

These preliminary findings suggest that KT applied using the “octopus” technique could serve as a tool that can be used in a controlled manner by trained healthcare professionals. Its use aims to generate further evidence to explore improved opportunities for assisting patients and potentially enhancing daily interventions.

### Study Limitations and Future Directions

This study has several limitations that should be acknowledged, including the absence of a control group and a small sample size, which preclude the establishment of causality. The limitation is the small number of cases, which restricts the ability to draw definitive conclusions from the findings. Nevertheless, this case series provides detailed clinical observations that may serve as a basis for future research. First, although patients received standard multidisciplinary care in the critical care setting, detailed information on concomitant interventions that may influence edema, such as diuretic therapy and nutritional management, was not systematically recorded as part of the nursing data collection. Therefore, these variables could not be controlled for analysis. Additionally, no specific adjunctive interventions aimed at directly reducing peripheral edema, such as compression therapy or manual lymphatic drainage, were implemented during the observation period. While this may allow a more focused clinical observation of the potential contribution of Kinesio taping within routine care, the multifactorial nature of edema in critically ill patients should be considered when interpreting the findings. Furthermore, nutritional status, including factors such as hypoalbuminemia, was not systematically assessed using validated tools, despite its potential influence on edema. Future studies with larger cohorts, longer follow-up periods, and standardized bandage replacement protocols, as well as comprehensive monitoring of these variables, are warranted to better control for potential confounding factors and to strengthen the validity and generalizability of the results.

Although the patients were under continuous clinical and hemodynamic monitoring in the ICU and no evidence of acute cardiac decompensation was observed, the absence of echocardiographic imaging limits the ability to fully exclude subclinical cardiovascular contributions to edema formation. On the other hand, inter-rater reliability was not formally assessed, as all extremity circumference measurements were intentionally performed by a single trained evaluator. This approach was adopted to enhance measurement consistency and minimize variability and potential bias associated with multiple evaluators in an intensive care setting.

However, the lack of formal reliability testing should be considered when interpreting the accuracy of the measurements. Despite these limitations, KT may represent a useful complementary strategy in the management of edema; however, its effects should be interpreted within the broader multifactorial clinical context characteristic of critically ill patients.

## 4. Conclusions

These preliminary findings indicate a potential for edema reduction in critically ill patients who are not receiving anticoagulant therapy, suggesting that KT could serve as an adjunctive tool in the treatment of edema in the Intensive Care Unit. Further studies with larger cohorts are needed to verify the effectiveness and safety of this intervention, considering each patient’s underlying pathologies to mitigate potential complications.

### Patient Perspectives

Case 1

“I noticed the change in the size and shape of my mother’s leg. I think the bandages do work and help”.

Case 2

“My feet felt very swollen; regular bandages and compression stockings burned. When the KT was applied, I felt relief and my feet were less swollen”.

Qualitative data from two cases indicated high treatment satisfaction and improved health-related quality of life. In Case 1, the patient’s family reported visible reduction in extremity volume, correlating with objective clinical measurements. In Case 2, the patient reported significant relief from previous discomfort caused by the conventional therapy (e.g., “burning sensations”) highlighting the superior skin tolerability and comfort of KT. These perspectives suggest that the intervention not only addresses physiological swelling but also enhances patient compliance by providing a more comfortable alternative to traditional compressive methods.

## Data Availability

The original data presented in the study are included in the article, further inquiries can be directed to the corresponding author.

## Abbreviations

The following abbreviations are used in this manuscript:

KT	Kinesio tape
ICU	Intensive Care Unit
BMI	Body Mass Index
DT	Distal third
MT	Medial third
PT	Proximal third
MARSI	Medical Adhesive-Related Skin Injury

## Appendix A

The following files are available as Appendix Materials: Table A1, Table A2, Table A3, Table A4, Table A5, Table A6, Table A7, Table A8 and Table A9: Detailed clinical, hemodynamic, and laboratory parameters for Cases 1, 2 and 3 during the intervention period.

**Table A1:** Vital Signs and Serum Electrolytes of Case 1 by Day of Treatment.

			Day		
Variable	1	2	3	4	5
HR (bpm)	86	78	91	82	73
RR (breaths/min)	18	16	16	20	19
Temperature (°C)	37.5	36.8	37.9	36.5	36
SpO_2_ (%)	91	93	90	92	91
BP (mmHg)	95/53	102/58	98/50	109/60	94/63
MAP (mmHg)	67	72	66	76	73
Glucose (mg/dL)	178	106	132	108	145
Na (mEq/L)	134	137	135	138	139
K (mEq/L)	4.8	4.5	4.2	4.5	4.5
Cl (mEq/L)	98	-	100	-	-
Mg (mEq/L)	2.4	-	2.7	-	-
P (mEq/L)	3.8	-	4.0	-	-
Ca (mEq/L)	8.6	-	8.7	-	-
Albumin g/dL	2.1	-	2.5	2.6	-

Notes: HR: heart rate; RR: respiratory rate; BP: blood pressure; MAP: mean arterial pressure; SpO_2_: oxygen saturation; Glucose: capillary blood glucose; Na: serum sodium; K: serum potassium; Cl: serum chloride; Mg: serum magnesium; P: serum phosphorus; Ca: serum calcium. -: data not determined.

**Table A2:** Skin and edema assessment of the treated left extremity of patient one.

			Day		
Variable	1	2	3	4	5
Intact extremity	+	+	+	+	+
Hydrated skin	+	-	+	+	+
Dehydrated (dry) skin	-	+	-	-	-
Ecchymosis	+	+	+	+	+
MARSI-type lesions	-	-	-	-	-
Inflammation	-	-	-	-	-
Pallor of the skin	+	+	-	+	+
Skin redness	-	-	+	-	-
Stemmer’s sign	+	+	+	+	-
Godet’s sign (+) to (++)	-	-	-	-	+
Godet’s sign (+++) to (++++)	+	+	+	+	-
Capillary refill < 3 s	-	+	+	+	+
Capillary refill > 4 s	+	-	-	-	-

Note: +: Present; -: Absent.

**Table A3:** General Aspects of the Treated Extremity of Patient 1.

			Day		
Variable	1	2	3	4	5
Cold extremity	+	+	+	-	+
Warm extremity	-	-	-	+	-
24-h fluid balance	+160 mL	+68 mL	+310 mL	+160 mL	+280 mL
Continuous mobilization	+	+	+	+	+
Feet elevated at 30°	-	-	-	-	-
Urine output > 0.5 mL/kg/hr	+	+	+	+	+
Urine output < 0.5 mL/kg/hr	-	-	-	-	-

Notes: +: Present; - Absent.

**Table A4:** Vital Signs and Serum Electrolytes of Case 2 by Day of Treatment.

			Day		
Variable	1	2	3	4	5
HR (bpm)	105	96	99	87	95
RR (breaths/min)	22	21	24	22	20
Temperature (°C)	37.3	37.1	37.5	37.2	37
SpO_2_ (%)	89	91	90	93	92
BP (mmHg)	100/60	110/60	110/60	110/50	120/60
MAP (mmHg)	73	76	76	70	80
Glucose (mg/dL)	125	117	138	122	98
Na (mEq/L)	133	138	136	136	134
K (mEq/L)	3.3	3.9	4.5	4.6	4.5
Cl (mEq/L)	96	-	100	-	-
Mg (mEq/L)	-	-	2.4	-	-
P (mEq/L)	-	-	-	-	-
Ca (mEq/L)	-	-	9.2	-	-
Albumin g/dL	3.1	-	-	2.8	-

Notes: HR: heart rate; RR: respiratory rate; BP: blood pressure; MAP: mean arterial pressure; SpO_2_: oxygen saturation; Glucose: capillary blood glucose; Na: serum sodium; K: serum potassium; Cl: serum chloride; Mg: serum magnesium; P: serum phosphorus; Ca: serum calcium; -: data not determined.

**Table A5:** Skin and edema assessment of both treated extremities of patient two.

			Day		
Variable	1	2	3	4	5
Intact extremity	+	+	+	+	+
Hydrated skin	+	+	+	+	+
Dehydrated (dry) skin	-	-	-	-	-
Ecchymosis	-	-	-	-	-
Marsi-type lesions	-	-	-	-	-
Inflammation	-	-	-	-	-
Pallor of the skin	+	+	-	-	-
Skin redness	-	-	+	+	+
Stemmer’s sign	-	-	-	-	-
Godet’s sign (+) to (++)	-	-	-	+	+
Godet’s sign (+++) to (++++)	+	+	+	-	-
Capillary refill < 3 s	+	+	+	+	+
Capillary refill > 4 s	-	-	-	-	-

Notes: +: Present; - Absent.

**Table A6:** General Aspects of Both Extremities by Day for Patient 2.

			Day		
Variable	1	2	3	4	5
Cold extremity	+	+	-	-	-
Warm extremity	-	-	+	+	+
24-h fluid balance	+430	+180	+260	+220	+420
Continuous mobilization	+	+	+	+	+
Feet elevated at 30°	-	-	+	-	-
Urine output > 0.5 mL/kg/hr	+	+	+	+	+
Urine output < 0.5 mL/kg/hr	-	-	-	-	-

Notes: +: Present; - Absent.

**Table A7:** Vital Signs and Serum Electrolytes of Case 3 by Day of Treatment.

			Day		
Variable	1	2	3	4	5
HR (bpm)	68	59	62	60	65
RR (breaths/min)	12	12	14	12	12
Temperature (°C)	35.7	36	36.2	35.9	36.3
SpO_2_ (%)	95	96	93	95	95
BP (mmHg)	125/65	118/60	115/70	121/68	130/72
MAP (mmHg)	85	79	85	85	91
Glucose (mg/dL)	140	126	121	149	138
Na (mEq/L)	142	140	141	142	142
K (mEq/L)	4.1	4.0	4.2	4.2	4.1
Cl (mEq/L)	106	99	98	100	103
Mg (mEq/L)	-	2.1	-	-	2.4
P (mEq/L)	-	3.7	-	-	4.0
Ca (mEq/L)	-	10.1	-	-	9.9
Albumin g/dL	-	3.5	-	-	2.9

Notes: HR: heart rate; RR: respiratory rate; BP: blood pressure; MAP: mean arterial pressure; SpO_2_: oxygen saturation; Glucose: capillary blood glucose; Na: serum sodium; K: serum potassium; Cl: serum chloride; Mg: serum magnesium; P: serum phosphorus; Ca: serum calcium; -: data not determined.

**Table A8:** Skin and edema assessment of both extremities treated of patient 3.

			Day		
Variable	1	2	3	4	5
Intact extremity	+	+	+	+	+
Hydrated skin	+	+	+	+	+
Dehydrated (dry) skin	-	-	-	-	-
Ecchymosis	-	-	-	-	-
Marsi-type lesions	-	-	-	-	-
Inflammation	-	-	-	-	-
Pallor of the skin	+	+	+	+	+
Skin redness	-	-	-	-	-
Stemmer’s sign	-	-	-	-	-
Godet’s sign (+) to (++)	+	+	+	+	+
Godet’s sign (+++) to (++++)	-	-	-	-	-
Capillary refill < 3 s	+	+	+	+	+
Capillary refill > 4 s	-	-	-	-	-

Notes: +: Present; - Absent.

**Table A9:** General Aspects of Both Extremities by Day for Patient 3.

			Day		
Variable	1	2	3	4	5
Cold extremity	-	-	-	-	-
Warm extremity	+	+	+	+	+
24-h fluid balance	+310	−150	+290	−240	−390
Continuous mobilization	+	+	+	+	+
Feet elevated at 30°	+	+	+	+	+
Urine output > 0.5 mL/kg/hr	+	+	+	+	+
Urine output < 0.5 mL/kg/hr	-	-	-	-	-

Notes: +: Present; - Absent.
